# Head injuries in a rural setup: Challenges and potential solutions

**DOI:** 10.4103/0974-2700.43202

**Published:** 2008

**Authors:** Amit Agrawal, Sudhakar R Joharapurkar, Keshav B Golhar, Vinay V Shahapurkar, Sankalp Dwivedi, Abhuday Meghe

**Affiliations:** Division of Neurosurgery, Datta Meghe Institute of Medical Sciences, Sawangi (Meghe), Wardha – 442 005, Maharashtra, India. E-mail: dramitagrawal@gmail.com

Sir,

Trauma in general, and head injury in particular, is the most frequent cause of morbidity, mortality, disability, and lost years of productive life in the population under 40–45 years of age.[1–3] To make matters worse, it has been estimated that about 1.3 billion people lack access to effective and affordable health care and, annually, an additional 150 million persons in 44 million households face financial catastrophe as a direct result of having to pay for health care. More than 100 million individuals are pushed into poverty by the need to pay for health services.[4] In this article we review our experience with trauma care, particularly head injuries, and try to find out the limitations and difficulties and the social situation in our area. Over a period of 16 months, a total of 575 patients were treated for head injury. The minimum age was 1 year and the maximum age was 84 years, with a mean of 33.06 years (SD 17.070). The majority of the patients had sustained mild head injuries. Surgical intervention was undertaken in 86 cases. The indications for surgery were intracranial hematomas, cerebral contusions, and depressed fractures. The mortality rate was 7.7%, mainly affecting patients with severe head injury. During this period we faced many difficulties in the management of the trauma cases; apart from those due to the severity of injury, there were problems due to the financial and social aspects of the cases. We tried to identify and highlight the limitations in the provision of health care and to find out the areas where further research could help to improve trauma care in rural areas, particularly in a developing country.

Any medical illness can lead to loss of employment and increase in expenditures; the expenses include that incurred for treatment and as well as that necessary to meet the needs of the family members who take care of the patient. Where the income of the patient or the family may be as low as 100 rupees/day (approximately US $2.50), it is difficult to understand how the costs of antibiotics, intensive care, and investigations can be met. The situation becomes especially difficult when the patient needs surgical intervention for a condition known to have a good outcome (e.g., extradural hematoma): we cannot deny the patient the benefit of the treatment. It is practically impossible to provide free treatment in situations where the resources are limited. Even if it were possible to waive the hospital charges, the cost of consumables has to be borne by the patient's relatives. When a severe head injury patient survives with some functional disability does he or she continues to be a burden on the family's resources? When the patient is the only earning member of the family the impact becomes uncertain and assessment becomes difficult”?

There are many questions that can be asked by relatives. For example, they may want to know if there are adequate facilities to manage the patient at the present facility, what the outcome is likely to be, or whether there is anything better by way of treatment available elsewhere; by far the most difficult question to answer is whether the patient can be transferred to the nearest higher center. When a patient with severe head injury is hemodynamically unstable or when there are associated life-threatening injuries, the decision on whether to transfer the patient can be especially difficult to make and, in spite of ventilatory support, the transportation may not be safe. All of us know that in more experienced hands there is a greater likelihood of the patient getting better. When you keep the patient, next day you find more friends and well wishers and in spite of best of the care lack of faith and confidence there is the mortality (because of the severity of the injury) than who will decide that the decision to the patient was correct. In spite of all kinds of pressures the dilemma of shifting the unstable patient will always confront us “would it be humane”?

The relatives of the patient may be aware of the advancements in care but may be unaware of the limitations in a given setup. While managing the patient in such a situation one must provide adequate counseling; the relatives should be informed of all the options available. Sometime one has to give in to the pressure and expectations and remind ourselves that we can not treat everybody. This should not allow us to leave the decision on the relatives, but lead us to help them assist to decide (i.e. need and urgency for surgical intervention in a large extradural hematoma or hemothorax). But before proceeding we have to win their confidence. Many questions will still remain, particularly when the facilities and resources are limited, and these include: How much we should push? How much responsibility must we shoulder?

The relatives are not totally ignorant; they expect adequate information and proper counseling. There is a need to develop a standardized surveillance system to keep track of head injury incidence, risk factors, causes, and outcomes; this information will be of use in the development of new and effective strategies for the evaluation and management of head injuries in the rural setup and aid in the identification of the predictors of outcome in such situations. Success and failure may not correlate in these situations with the percentage of positive achievements, however good outcome has helped and helping us to win the confidence of people.
